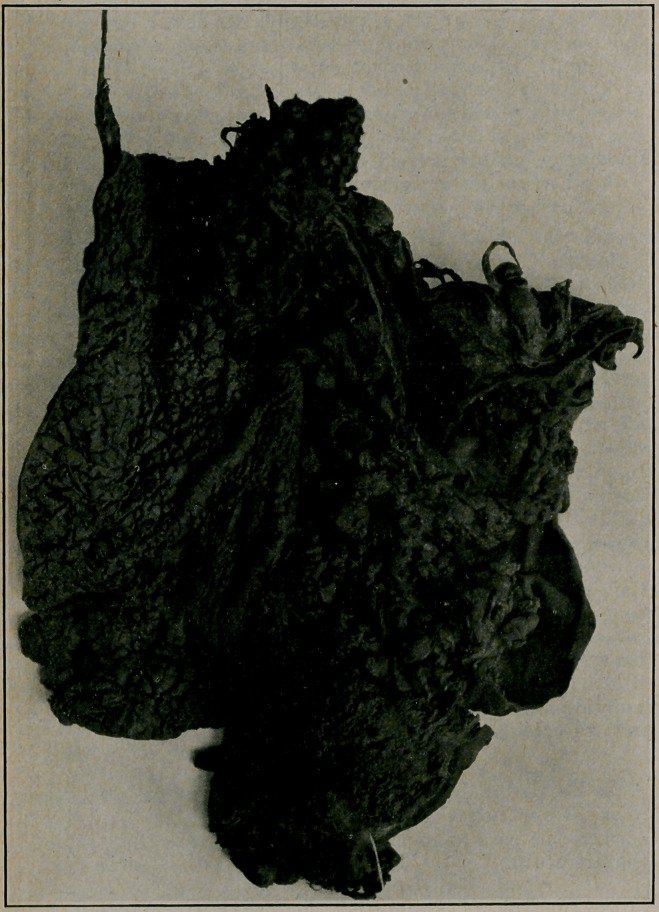# Polyposis Gastrica (Polyadenoma)

**Published:** 1914-08

**Authors:** 


**Polyposis Gastrica (Polyadenoma),** D. S. Lamb of Washington in Wash. Med. Annals, May, 1914. In 1868, Dr. D. H. Browner, Acting Asst. Surg. U. S. A. at Richmond, sent to the Army Medical Museum, a specimen of a stomach from a freedman aged 30. The principal symptoms were emaciation, jaundice and vomiting. At necropsy, the heart and lungs were normal, the stomach contained coffee ground material and the diaphragm was studded with minute nodules. At the time, the case was considered villous cancer. Recent examination by Dr. E. R. Whitmore has established the diagnosis of polyposis there being no epithelial tissue in the nodules. Reference is made to the article of the late J. S. Myer of St. Louis in the Jour. A. M. A., Nov. 29, 1913.

## Figures and Tables

**Figure f1:**